# Supplementary material for: The Extracts of Polygonum cuspidatum Root and Rhizome Block the Entry of SARS-CoV-2 Wild-Type and Omicron Pseudotyped Viruses via Inhibition of the S-Protein and 3CL Protease
Source: Molecules. 2022 Jun 13;27(12):3806. doi: 10.3390/molecules27123806 (PMC9231230; doi:10.3390/molecules27123806)
Supplement: Supplementary file 1 [file molecules-27-03806-s001.zip › molecules-1750702-supplementary.pdf]

The Extracts of *Polygonum cuspidatum* Root and Rhizome Block the Entry of SARS-CoV-2 Wild-Type and Omicron Pseudotyped Viruses via Inhibition of the S-Protein and 3CL Protease

Shengying Lin [1,2], Xiaoyang Wang [1,2], Roy Wai-Lun Tang [1,2], Hung Chun Lee [1,2], Ho Hin Chan [2], Sheyne S.A. Choi [2], Tina Ting-Xia Dong [1,2], Ka Wing Leung [1,2], Sarah E. Webb [2], Andrew L. Miller [2] and Karl Wah-Keung Tsim [1,2,*]

[1] Division of Life Science and Centre for Chinese Medicine, The Hong Kong University of Science and Technology, Hong Kong, China; lishlin@ust.hk (S.L.); wangxiaoyang@ust.hk (X.W.); roytwl@ust.hk (R.W.-L.T.); leoleehc@ust.hk (H.C.L.); botina@ust.hk (T.T.-X.D.); lkwing@ust.hk (K.W.L.); botsim@ust.hk (K.W.-K.T.)
[2] State Key Laboratory of Molecular Neuroscience, The Hong Kong University of Science and Technology, Hong Kong, China; hhbchan@hku.hk (H.H.C.); ssachoi@ust.hk (S.S.A.C.); barnie@ust.hk (S.E.W.); almiller@ust.hk (A.L.M.)
* Correspondence: botsim@ust.hk (K.W.-K.T.); Tel.: +852-2358-7332

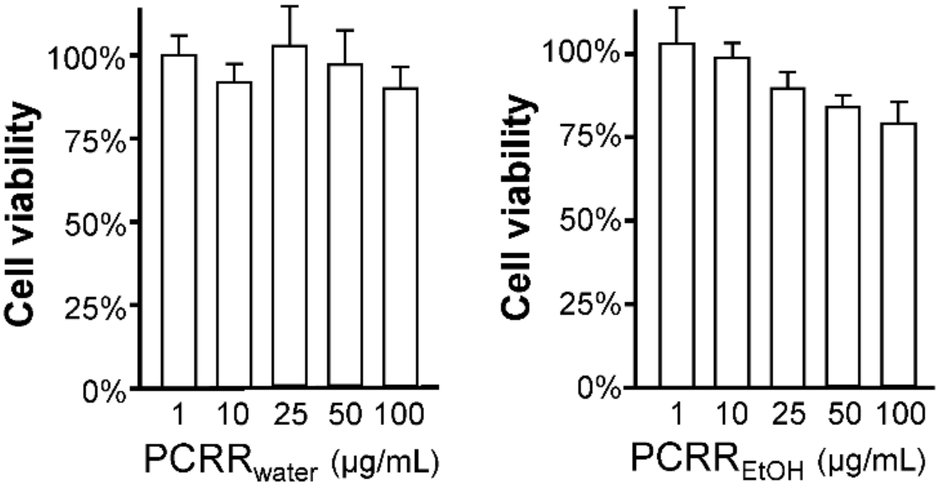

Figure S1. Effect of the PCRR extracts on the viability of HEK293T-ACE2 cells, measured with the MTT assay. $PCRR_{water}$ and $PCRR_{EtOH}$ were tested at concentrations of 1, 10, 25, 50 and 100 μg/mL, before the inclusion of MTT solution at 20 μL/well to give a final concentration of 0.5 mg/mL. The optical density of each well was determined at 492 nm. Cell viability was expressed as a percentage of an untreated control. All data are shown as mean ± SD, *n* = 3.

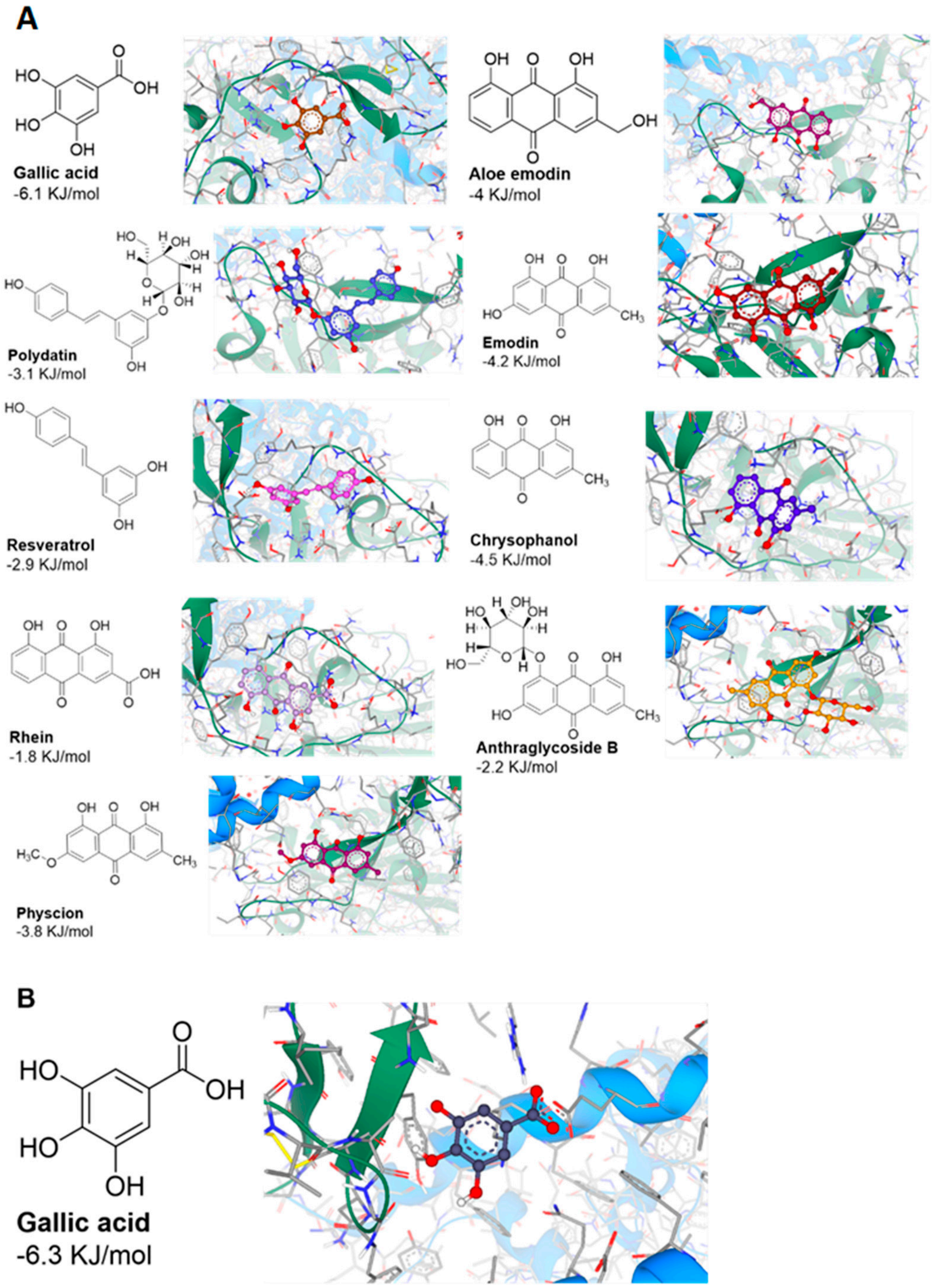

Figure S2. Analysis of binding of the PCRR phytochemicals to the receptor binding domain (RBD) of the S-protein. (**A**) A computational docking analysis indicated that residues 438-506 of the S-protein (PDB code: 6LZG) from the wild-type virus, were the receptor binding domain. The structures of the phytochemicals were downloaded from PubChem (https://pubchem.ncbi.nlm.nih.gov/ accessed on 1 May 2022). **(B)** Residues 438-506 of the S-protein from the omicron variant (PDB code: 7T9L) were also considered to be the binding site. In each case, the proposed binding energy (in KJ/mol) is indicated.
